# Avian malaria alters the dynamics of blood feeding in *Culex pipiens* mosquitoes

**DOI:** 10.1186/s12936-019-2690-5

**Published:** 2019-03-15

**Authors:** Stéphane Cornet, Antoine Nicot, Ana Rivero, Sylvain Gandon

**Affiliations:** 1 Centre d’Ecologie Fonctionnelle et Evolutive (CEFE), UMR CNRS 5175, Montpellier, France; 2 MIVEGEC (CNRS, Université de Montpellier- IRD), Montpellier, France

**Keywords:** *Plasmodium relictum*, Malaria, *Culex pipiens*, Blood feeding, Vector behaviour

## Abstract

**Background:**

Some *Plasmodium* species have the ability to modify the behaviour of their mosquito vectors. This is thought to be an adaptive strategy that maximizes the parasite’s transmission.

**Methods:**

The effect of *Plasmodium relictum* infections on the blood feeding behaviour of *Culex pipiens quinquefasciatus* mosquitoes was monitored.

**Results:**

*Plasmodium* infections did not alter the proportion of blood fed mosquitoes but they did affect the dynamics and the size of the blood meal. Sporozoite-infected mosquitoes completed their blood meal 1.3 times later than uninfected mosquitoes and ended up with smaller blood meals.

**Conclusion:**

The potential adaptive nature of this manipulation of mosquito behaviour is discussed in the light of previous studies on other malaria models.

**Electronic supplementary material:**

The online version of this article (10.1186/s12936-019-2690-5) contains supplementary material, which is available to authorized users.

## Background

Malaria transmission is tied to the behaviour of its insect vectors [1–3]. Several studies have shown how *Plasmodium* parasites have evolved strategies to modify the foraging and feeding behaviour of their mosquito vectors in order to enhance malaria transmission [1–3]. This adaptive manipulation of the behaviour of mosquitoes may take place at two different steps of the malaria life cycle. First, parasite manipulation may turn infected hosts into more attractive targets for uninfected vectors [4–6]. Indeed, several studies found that uninfected mosquitoes show a preference for feeding on infected hosts (but see contradictory results obtained by [7] and [8]). This preference has been shown for human [9], avian [10, 11], and rodent *Plasmodium* parasites [12]. Second, parasites may also manipulate the behaviour of infected vectors [4–6]. For instance, malaria-infected *Anopheles* mosquitoes express enhanced attraction to human odours [13] and are more likely to approach and to attempt to feed on humans than uninfected ones [14]. In addition, after landing on the host, mosquitoes infected with the transmissible parasite stages of *Plasmodium* have been shown to exhibit higher host probing and biting rates and/or longer blood meals [14–19]. These studies illustrate how a better knowledge of the biting behaviour of the vector can have important consequences for the epidemiological dynamics of vector-borne diseases [6, 20].

In recent years, avian malaria has emerged as a unique animal model for understanding the ecology and evolution of *Plasmodium* parasites [21]. Previous work carried out using the natural *Culex pipiens* sensu lato-*Plasmodium relictum* mosquito-parasite combination has shown that parasite infection increases the attractiveness of birds to both uninfected and infected mosquitoes [10]. Note however that contradictory results have been obtained by [22] and [23]. Unfortunately, it is difficult to pinpoint the reason for this discrepancy because different experimental protocols and/or host species were used. The present study explores another manipulative trait of avian *Plasmodium* parasites: their ability to modify the feeding behaviour of mosquitoes. In particular, this work monitors whether mosquitoes infected with the transmissible (sporozoite) stages of *Plasmodium* differ from their uninfected counterparts in: (i) the probability of feeding on an uninfected bird, (ii) the dynamics of the blood feeding, i.e. the time they require to find the host and complete the blood feeding bout, and (iii) the size of the blood meal ingested. For this purpose, uninfected and sporozoite-infected female *Culex pipiens* were paint-marked and given the opportunity to blood feed on uninfected birds. The cages were visited regularly and all resting, blood-fed mosquitoes removed, their infection status recorded and the size of the blood meal ingested quantified.

This experimental set up was used to test three predictions following from a scenario of *Plasmodium* manipulation of mosquito behaviour: First, all else being equal, sporozoite-infected mosquitoes are expected to be more motivated to blood feed than their uninfected counterparts. As a result, the blood feeding success (total proportion of blood fed mosquitoes at the end of the experiment) may be higher for sporozoite-infected than for uninfected mosquitoes. Second, a higher motivation to blood feed may also translate into faster blood feeding dynamics (the time required to initiate and complete the blood feeding bout). Third, sporozoite-infected mosquitoes are expected to obtain smaller blood meals and therefore lead to non-satiated mosquitoes which are, in turn, expected to be more motivated to look for alternative hosts [24].

## Methods

### Malaria parasites and birds

*Plasmodium relictum* (lineage SGS1) is the most prevalent form of avian malaria parasite in Europe [21] and was used to infect the canary birds and the mosquitoes in the present study. This generalist *Plasmodium* parasite lineage was originally isolated from wild house sparrows and maintained in the laboratory via subsequent passages to naïve canaries by intraperitoneal injection of infected blood or by completing the parasite cycle through mosquitoes [25]. Mosquitoes of the *Culex pipiens* complex are the main vectors of *P. relictum* in the field [26, 27].

Prior to the onset of the experiment, 1-year old domestic canaries (*Serinus canaria*) were screened to be free from any haemosporidian infection [10, 11]. Birds used for mosquito infection were experimentally infected by intraperitoneal injection of ca. 50–100 µL of infected blood from an infected canary stock. Infection success was monitored 10 days post inoculation using thin blood smears.

### Infected and uninfected mosquitoes

Experiments were conducted with a laboratory strain of *Culex pipiens quinquefasciatus* (SLAB) [11]. Mosquitoes were reared under standard conditions [28]. Infected and uninfected mosquitoes were obtained as previously described in [11]. Seven cages, each containing around 120 female mosquitoes (6–7 day old after emergence), were set up. Distinct canary birds were used to blood feed mosquitoes from each of the seven mosquito cages. Four cages were provided with an infected canary, while the three other cages were provided with an uninfected one. The four infected birds were inoculated with the parasite 12 days earlier following standard laboratory procedures, and were thus in the acute phase of the infection [25]. Previous work has shown that this protocol ensures that > 90% of the mosquitoes become infected [28]. Unfed mosquitoes were discarded. Four days after the infected or uninfected blood meal (day 4 post blood meal, pbm), and until the beginning of the behavioural assay, cages were provided with a water-filled plastic tray to allow females to lay their eggs. On day 7 pbm, a sample of five mosquitoes were haphazardly collected from each of the seven cages and dissected to monitor the presence of oocysts in the midgut.

### Experimental design

The experiment was carried out 13 days pbm [11], when *Plasmodium* has reached the salivary glands (sporozoite stage), and can be transmitted to novel hosts. Given that *P. relictum* sporogony is asynchronous, some midgut oocysts may, however, still be developing at this stage [29]. Four days before the assays, mosquitoes were marked using small amount of either pink or yellow fluorescent powder applied as a dust storm [11]. The two colours were used in rotation to mark uninfected and infected mosquitoes to avoid a potential colour effect.

To minimize host defensive behaviours that may alter the mosquito feeding process during the assay, birds were immobilized in a specially designed PVC tube that rendered their legs accessible to the mosquitoes while protecting the rest of the body from the bites [10]. Six uninfected birds were used for the experiment. Each bird was placed inside a cage (dimensions L40 × W30 × H30 cm) with around 45 uninfected and 45 sporozoite-infected mosquitoes. Each batch of infected and uninfected mosquitoes contained a similar proportion of mosquitoes from the 4 infected and 3 uninfected cages, respectively.

Mosquitoes were allowed to feed for 3 h (from 7 to 10 pm). Every 10 min, blood-fed mosquitoes were retrieved from the cages using an aspirator and stored in separate plastic vials. Unfed mosquitoes were also collected at the end of the experiment. The numbers of sporozoite-infected and uninfected mosquitoes (assessed upon the observation of the colour powder under the binoculars) were determined for each sampling time-point. Samples were frozen until the blood meal quantification assay.

### Blood meal size

The amount of blood ingested by the mosquitoes was determined using a colorimetric assay to quantify the concentration of haemoglobin in the sample following the protocol described by Briegel et al. [30]. Prior to the assay, a wing was collected and its length was measured (using Image J software) to get a proxy of the size of mosquitoes. Individual mosquitoes were dissected and their whole abdomens crushed in 0.5 mL of Drabkin’s solution. The samples were incubated for 20 min at room temperature. Then, 0.5 mL chloroform was added and samples were centrifuged for 5 min at 6000 rpm. The supernatant (200 µL) was collected and the amount of haemoglobin in the solution was estimated by the optical density measured in a spectrophotometer at 540 nm.

### Statistical analyses

The analyses were carried out using R (v. 3.4.1). Blood feeding success was analyzed using a generalized linear mixed-model (package lme4, *glmer* function, binomial distribution) and the dynamics of blood feeding was analysed using a Cox proportional hazards mixed effect model (package coxme, *coxme* function) with unfed mosquitoes applied as a censor. In this case, the hazard function is the instantaneous rate of change in the log number of blood-fed mosquitoes per unit of time [31]. Hazard ratio (HR) was obtained from the model as an estimate of the ratio between the instantaneous change in blood-feeding success between the infected and uninfected groups. Models were fitted by specifying mosquito infection status (infected, uninfected) as a fixed effect and the bird identity as a random effect. Model significance was tested by comparing the change in deviance with and without the term using a χ^2^ distribution. Estimates of median feeding time (and confidence intervals) were obtained using survival fits (package survival, *survfit* function).

Blood meal sizes (OD values) were analysed using linear mixed-models (package nlme, *lme* function, normal distribution). Models were fitted with mosquito infection status, size and their interaction as fixed effects, and the bird identity as a random effect. Models were simplified by sequentially eliminating non-significant terms (*P* > 0.05) to obtain minimal adequate models using a standard procedure of likelihood comparison (using the function anova.lme specifying a marginal type test). Significant *P* values in the text are for the minimal models whereas non-significant values refer to those obtained before the deletion of the term from the model. Three individuals with negative OD values for the blood meal assay had been removed from the data set.

## Results

The dissection of mosquitoes on day 7 pbm revealed that all mosquitoes that fed on infected birds had at least one oocyst (see Additional file 1: Table S1). There were no significant differences in size between infected and uninfected mosquitoes (wing length infected: 3306 ± 9 µm, uninfected: 3316 ± 9 µm; *F*_1, 402_ = 0.63, *P* = 0.4284).

The blood feeding success (proportion of mosquitoes that obtained a blood meal after the 3 h period) did not depend on whether mosquitoes were infected (mean ± se: 0.78 ± 0.03) or uninfected (0.83 ± 0.04; $\chi^{ 2}_{ 1}$ = 1.96, *P* = 0.1617). However, the analysis of the proportion of blood-fed mosquitoes after 1 h revealed that uninfected mosquitoes had a significantly higher blood feeding success than their uninfected counterparts (uninfected: 0.46 ± 0.04, infected: 0.31 ± 0.03; $\chi^{ 2}_{ 1}$ = 12.4, *P* < 10^−3^). The contrast between the results after 1 h and 3 h suggests that infected and uninfected mosquitoes did not feed at the same speed.

**Fig. 1:**
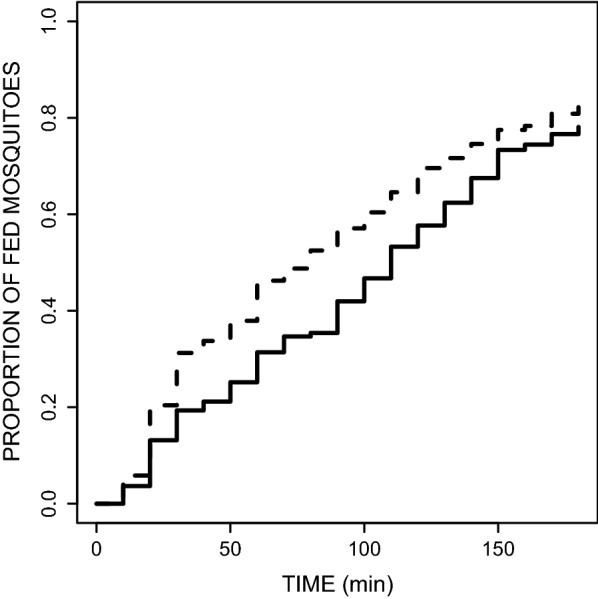
Effect of the status of infection by *Plasmodium relictum* of mosquitoes [infected by sporozoites (solid line) vs uninfected (dashed line)] on the temporal dynamics of mosquito blood feeding. Around 90 *Culex pipiens* mosquitoes (45 uninfected and 45 sporozoite-infected mosquitoes) were presented an uninfected birds on which they were allowed to feed. Every 10 min, blood-fed mosquitoes were taken out from the cage and sorted according to their status of infection. The experiment was replicated 6 times (using 6 different uninfected birds, see Additional file 2: Figure S1 showing the blood feeding dynamics on each bird)

Indeed, *P. relictum* infection has a significant effect on the blood feeding dynamics (HR ± s.e. = 0.26 ± 0.098, $\chi^{ 2}_{ 1}$ = 6.93, *P* = 0.0083; Fig. 1). On average, uninfected mosquitoes completed their blood meal, i.e. were found blood fed and resting on the walls of the cage, significantly faster than their sporozoite-infected counterparts (median times [and 95% confidence intervals]; uninfected: 80 min [60,90], infected: 110 min [100,120]).

**Fig. 2:**
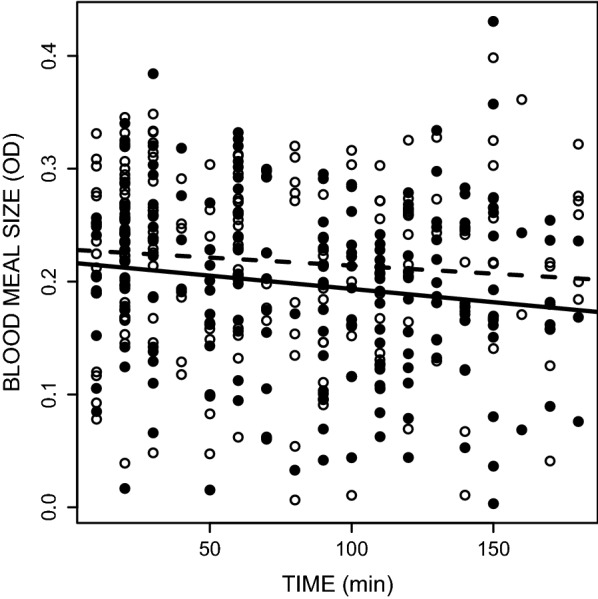
Effect of the status of infection by *Plasmodium relictum* (infected by sporozoites (solid line and dots) *vs* uninfected (dashed line and empty dots)) on the blood meal size of *Culex pipiens* mosquitoes across time. The amount of ingested blood was estimated by quantifying the concentration of haemoglobin in the sample using a spectrophotometric assay (optical density values). Each dot refers to the estimated blood meal size of a blood-fed mosquito taken out of the experimental cage. The experiment was replicated 6 times (using 6 different uninfected birds, see Additional file 3: Figure S2 showing the blood feeding dynamics on each bird)

*Plasmodium*-infected mosquitoes took smaller blood meals (OD 0.197 ± 0.005) than their uninfected counterparts (OD 0.218 ± 0.005) (infection effect *F*_1,400_ = 6.46, *P* = 0.011; Fig. 2). Mosquito size explained part of the variation in the blood meal size (*F*_1,400_ = 8.34 *P* = 0.004) with bigger mosquitoes taking a larger amount of blood. Interestingly, in both infected and uninfected mosquitoes, the size of the blood meal decreased with time, i.e. mosquitoes that fed later also took smaller blood meals (time effect *F*_1,400_ = 5.73, *P* = 0.017; Fig. 2).

## Discussion

These results show that the infection of *Culex pipiens quinquefasciatus* by *P. relictum* does not influence the proportion of blood fed mosquitoes retrieved at the end of this experiment. Both sporozoite-infected (79%) and uninfected mosquitoes (83%) were as likely to take a blood meal during the 3 h experiment (Fig. 1). Interestingly, however, a very different conclusion would have been reached had the experiment been run for 1 h. Indeed, since on average uninfected mosquitoes completed their blood meal 1.3 times faster than their sporozoite-infected counterparts, at the end of the first hour amongst the blood fed mosquitoes there were significantly more uninfected mosquitoes than uninfected ones. These results highlight the critical impact that different experimental protocols can have on the conclusions obtained from this type of behavioural studies and may explain some of the contradictory results obtained in studies aiming to investigate the manipulation of host-choice behaviour by *Plasmodium* parasites [7, 8, 22, 23].

There are several potential explanations for the significant impact of a *Plasmodium* infection on the dynamics of mosquito blood-feeding behaviour. The measurement of blood feeding dynamics compounded several different events: the off-host behaviour (the time required to locate the host and the ‘motivation’ to subsequently feed on it) and the on-host behaviour (the time required to probe and complete the blood meal). Each of these steps could have been negatively, and potentially independently, affected by the parasite infection. Regarding the off-host behaviour, infected mosquitoes may have taken longer to locate the host and may thus have started their feeding bout later than uninfected mosquitoes. If so, the lowered host seeking performance of infected mosquitoes would not be an adaptive manipulative trait of the parasite (as such an impaired host-seeking would hamper its transmission), but rather an expression of its virulence (i.e. a by-product of the infection).

Alternatively, uninfected and infected mosquitoes may have been equally efficient or ‘motivated’ to feed on the host, but infected mosquitoes may have taken longer probing and ingesting the blood (on-host behaviour). Indeed, the presence of sporozoites in the salivary glands of infected mosquitoes has been shown to reduce the production of apyrase: a salivary enzyme that plays a key role in the blood feeding process of many haematophagous insects, including mosquitoes [32]. The apyrase inhibits ADP-dependent platelet aggregation (haemostasis) thereby shortening the blood probing behaviour and facilitating blood intake. Previous work has shown that infection by *Plasmodium gallinaceum* in *Aedes aegypti* leads to a one-fourth reduction of the apyrase activity in the saliva [15], resulting in multiple probing (twice more attempts in infectious than uninfected mosquitoes) and prolonged vector-host contact [14, 15]. The hypothesis that *P. relictum* may act on apyrase activity is also consistent with the observation that *Plasmodium*-infected mosquitoes take smaller blood meals (Fig. 2). However, blood meal size is also explained by the timing of the blood meal: mosquitoes that feed later also take somewhat smaller blood meals (Fig. 2). This slight, albeit statistically significant, negative relationship between blood meal size and time could also partially explain the smaller blood meals of infected mosquitoes, since they fed, on average, later than their uninfected counterparts. The reasons for the decrease in blood meal size with time remain to be determined, but may be due to a dynamic host response to the continued exposure to the mosquito saliva, including changes in haemostasis (blood coagulation, platelet aggregation and vasoconstriction) and immunity [33].

Additional behavioural, physiological and olfactory experiments are needed to confirm which step of the mosquito behaviour is affected by the infection (off-host or on-host behaviour) and to identify the mechanisms underlying these behavioural modifications. This will go a long way towards establishing whether these behavioural modifications result from parasite adaptations or whether they are simply triggered by a general response to an immune challenge [34–36]. Analysis of the costs and benefits of this behavioural change are also needed to evaluate their implication on the epidemiology and evolution of the parasite [6, 37, 38].

## Conclusion

This study shows that *Plasmodium relictum* infections affect the dynamics of the blood feeding behaviour of *Culex pipiens quinquefasciatus* mosquitoes. Sporozoite-infected mosquitoes completed their blood meal later than uninfected mosquitoes and ended up with smaller blood meals. At first sight, this effect may seem maladaptive for the parasite, but a slower ability to take up a blood meal may result from a modification of the ability of the infected mosquito to blood feed and could result in higher transmission rates. Additional experiments are needed to confirm which step of the mosquito behaviour is affected by the infection. The adaptive nature of these behavioural modifications requires a careful evaluation of the fitness costs and benefits for the parasite.

## Additional files

**Additional file 1: Table S1.** Infection status of the 7 birds used to feed the mosquitoes in the first stage of the experiment. The dissection of a few mosquitoes (number of oocysts/mosquito indicated in the final column) confirmed that most of the mosquitoes that fed on infected birds became infected, while none of the mosquitoes that fed on uninfected birds did. In the second stage of the experiment, mosquitoes that fed on infected (uninfected) birds were pooled and color painted. The blood feeding behavior of these mosquitoes (on 6 uninfected birds) was monitored in the second stage of the experiment.

**Additional file 2: Figure S1.** Effect of the status of infection by *Plasmodium relictum* of mosquitoes (infected by sporozoites (solid line) vs uninfected (dashed line)) on the temporal dynamics of mosquito blood feeding (same as in Fig. 1 but for each of the 6 birds used in the experiment).

**Additional file 3: Figure S2.** Effect of the status of infection by *Plasmodium relictum* (infected by sporozoites (solid line and dots) vs uninfected (dashed line and empty dots)) on the blood meal size of Culex pipiens mosquitoes across time (same as in Fig. 2 but for each the 6 birds used in the experiment).

**Additional file 4.** Data indicates the time at which uninfected and sporozoite-infected mosquitoes completed their blood meal (replicated on 6 uninfected birds).

**Additional file 5.** Data indicates the blood meal size of blood fed mosquitoes (optical density values).
