# Supplementary material for: Avian malaria alters the dynamics of blood feeding in Culex pipiens mosquitoes
Source: Malar J. 2019 Mar 15;18:82. doi: 10.1186/s12936-019-2690-5 (PMC6420798; doi:10.1186/s12936-019-2690-5)
Supplement: Supplementary file 1 — Additional file 1: Table S1. Infection status of the 7 birds used to feed the mosquitoes in the first stage of the experiment. The dissection of a few mosquitoes (number of oocysts/mosquito indicated in the final column) confirmed that most of the mosquitoes that fed on infected birds became infected, while none of the mosquitoes that fed on uninfected birds did. In the second stage of the experiment, mosquitoes that fed on infected (uninfected) birds were pooled and color painted. The blood feeding behavior of these mosquitoes (on 6 uninfected birds) was monitored in the second stage of the experiment. [file 12936_2019_2690_MOESM1_ESM.docx]

**Table S1 :** Infection status of the 7 birds used to feed the mosquitoes in the first stage of the experiment. The dissection of a few mosquitoes (number of oocysts/mosquito indicated in the final column) confirmed that most of the mosquitoes that fed on infected birds became infected, while none of the mosquitoes that fed on uninfected birds did. In the second stage of the experiment, mosquitoes that fed on infected (uninfected) birds were pooled and color painted. The blood feeding behavior of these mosquitoes (on 6 uninfected birds) was monitored in the second stage of the experiment.

| **Birds** | | **Parasitaemia (%)** | **Gametocytemia (%)** | **Oocytes** |
| --- | --- | --- | --- | --- |
| **Infected** | Red 9LXW | 0.5 | 0.14 | {1,1,2,2,2} |
|  | Bleu 305 | 0.9 | 0.15 | {1,1,3,6,11} |
|  | Argent 14 | 0.82 | 0.13 | {3,4,6,11,12} |
|  | Orange 11 | 20.4 | 4 | {3,19,31,40,244} |
| **Uninfectd** | Rouge 18 | 0 | 0 | {0,0,0,0,0} |
|  | Orange 12 | 0 | 0 | {0,0,0,0,0} |
|  | Violet 12 | 0 | 0 | {0,0,0,0,0} |
