# Supplementary figures and images for: Avian malaria alters the dynamics of blood feeding in Culex pipiens mosquitoes
Source: Malar J. 2019 Mar 15;18:82. doi: 10.1186/s12936-019-2690-5 (PMC6420798; doi:10.1186/s12936-019-2690-5)

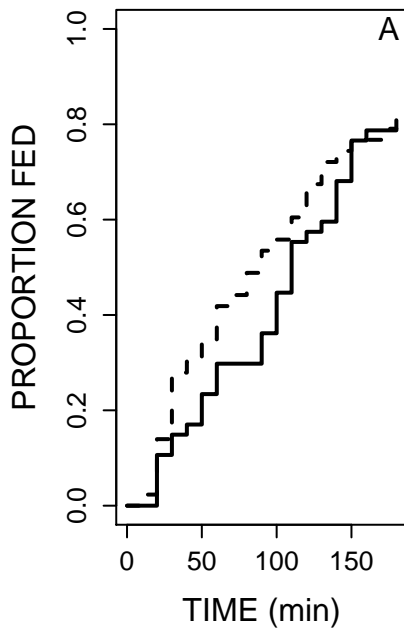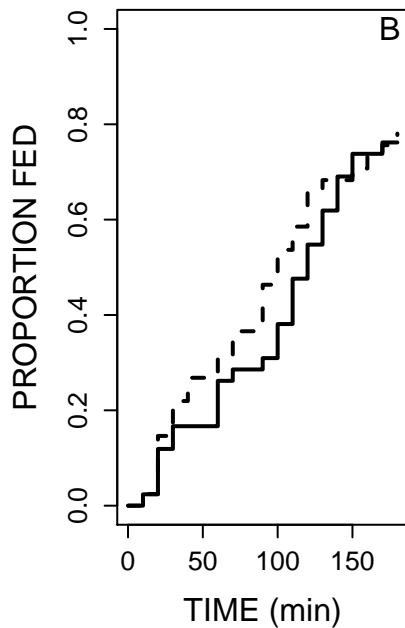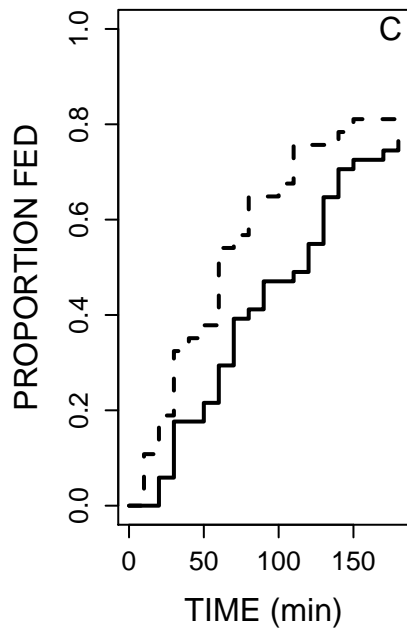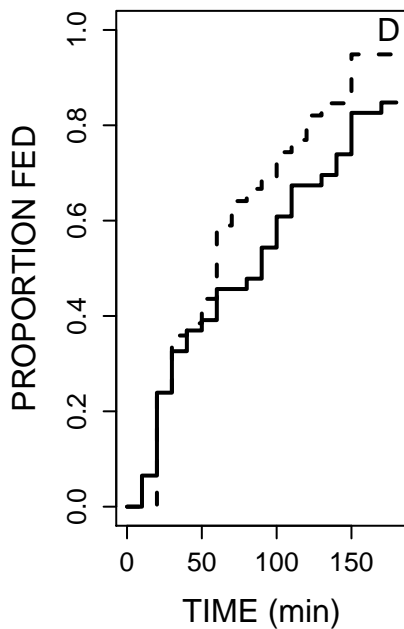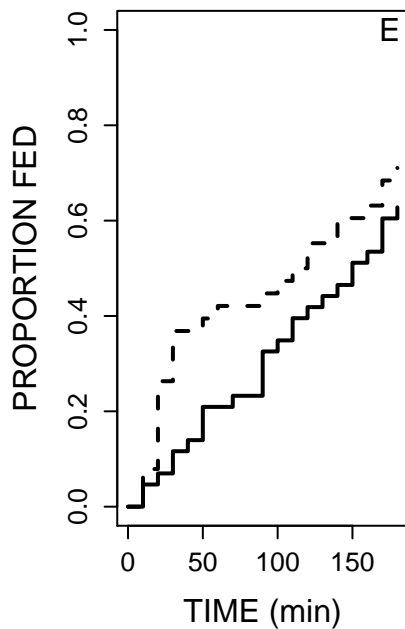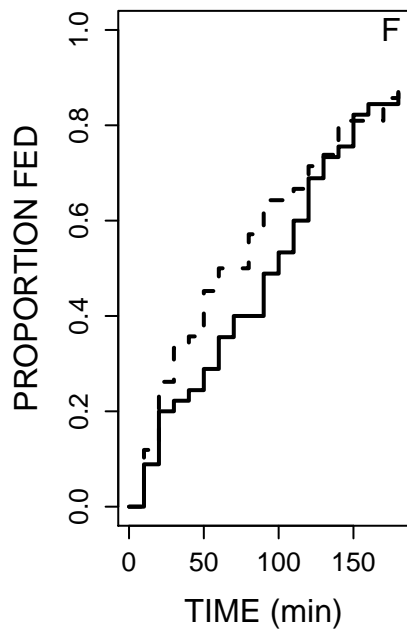

Supplement: Supplementary file 2 — Additional file 2: Figure S1. Effect of the status of infection by Plasmodium relictum of mosquitoes (infected by sporozoites (solid line) vs uninfected (dashed line)) on the temporal dynamics of mosquito blood feeding (same as in Fig. 1 but for each of the 6 birds used in the experiment). [file 12936_2019_2690_MOESM2_ESM.pdf]

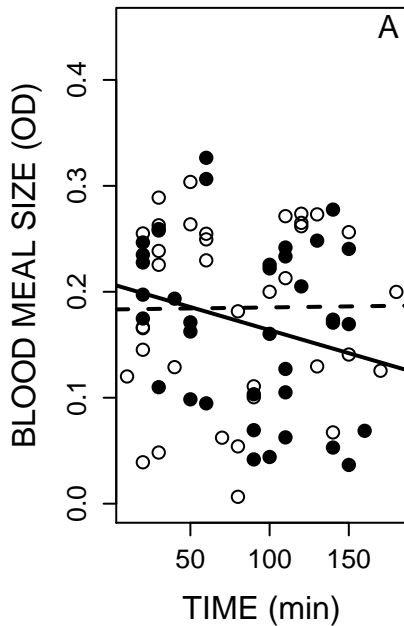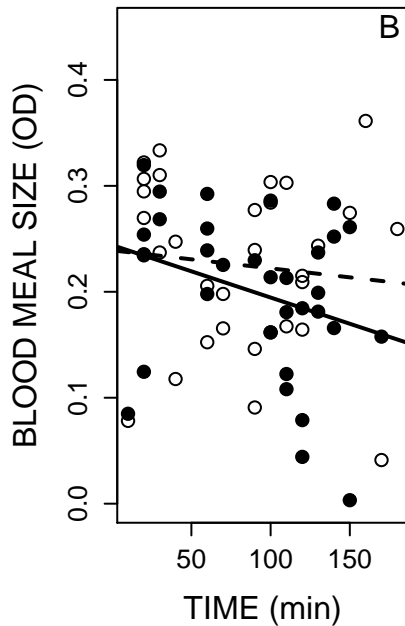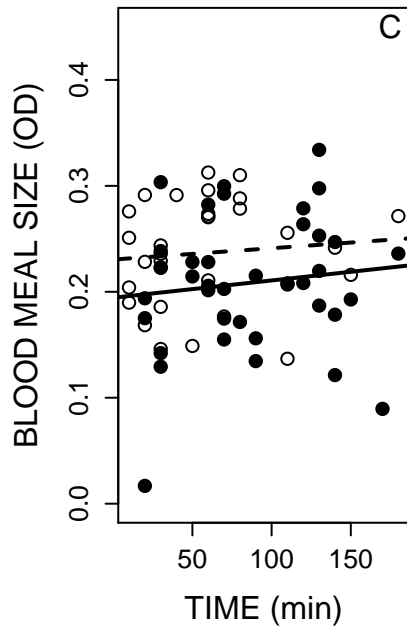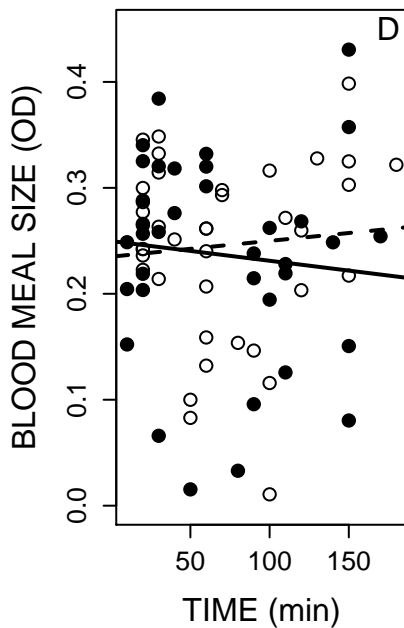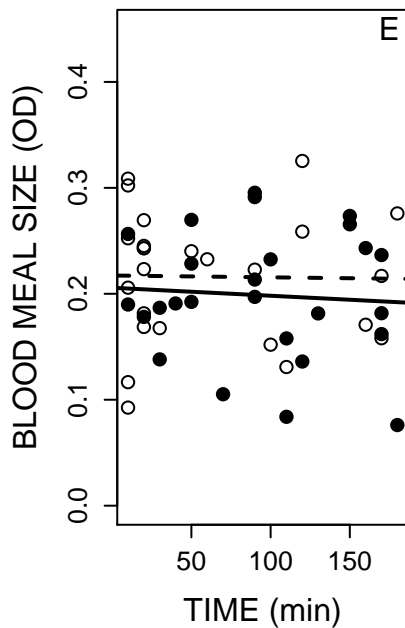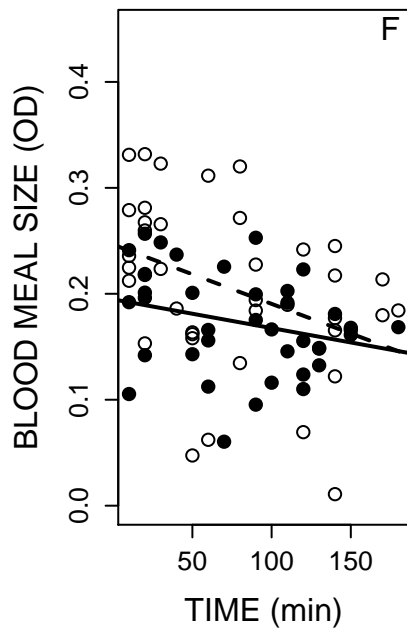

Supplement: Supplementary file 3 — Additional file 3: Figure S2. Effect of the status of infection by Plasmodium relictum (infected by sporozoites (solid line and dots) vs uninfected (dashed line and empty dots)) on the blood meal size of Culex pipiens mosquitoes across time (same as in Fig. 2 but for each the 6 birds used in the experiment). [file 12936_2019_2690_MOESM3_ESM.pdf]
